# Improving Standards of Care in Obstructed Labour: A Criteria-Based Audit at a Referral Hospital in a Low-Resource Setting in Tanzania

**DOI:** 10.1371/journal.pone.0166619

**Published:** 2016-11-28

**Authors:** Andrew H. Mgaya, Hussein L. Kidanto, Lennarth Nystrom, Birgitta Essén

**Affiliations:** 1 Department of Obstetrics and Gynaecology, Muhimbili National Hospital, Dar es Salaam, Tanzania; 2 Department of Women’s and Children’s Health/International Maternal and Child Health, Uppsala University, Uppsala, Sweden; 3 Reproductive and Child Health section, Ministry of Health, Community Development, Gender, Elderly and Children, Dar es Salaam, Tanzania; 4 Department of Public Health and Clinical Medicine, Epidemiology and Global Health, Umeå University, Umeå, Sweden; Leibniz Institute for Prvention Research and Epidemiology BIPS, GERMANY

## Abstract

**Objective:**

In low-resource settings, obstructed labour is strongly associated with severe maternal morbidity and intrapartum asphyxia, and consequently maternal and perinatal deaths. This study evaluated the impact of a criteria-based audit of the diagnosis and management of obstructed labour in a low-resource setting.

**Methods:**

A baseline criteria-based audit was conducted from October 2013 to March 2014, followed by a workshop in which stakeholders gave feedback on interventions agreed upon to improve obstetric care. The implemented interventions included but were not limited to introducing standard guidelines for diagnosis and management of obstructed labour, agreeing on mandatory review by specialist for cases that are assigned caesarean section, re-training and supervision on use and interpretation of partograph and, strengthening team work between doctors, mid-wives and theatre staff. After implementing these interventions in March, a re-audit was performed from July 2015 to November, 2015, and the results were compared to those of the baseline audit.

**Results:**

Two hundred and sixty deliveries in the baseline survey and 250 deliveries in the follow-up survey were audited. Implementing the new criteria improved the diagnosis from 74% to 81% (*p* = 0.049) and also the management of obstructed labour from 4.2% at baseline audit to 9.2% at re-audit (*p* = 0.025). Improved detection of prolonged labour through heightened observation of regular contractions, protracted cervical dilatation, protracted descent of presenting part, arrested cervical dilation, and severe moulding contributed to improved standards of diagnosis (all *p* < 0.04). Patient reviews by senior obstetricians increased from 34% to 43% (*p* = 0.045) and reduced time for caesarean section intervention from the median time of 120 to 90 minutes (*p* = 0.001) improved management (all *p* < 0.05). Perinatal outcomes, neonatal distress and fresh stillbirths, were reduced from 16% to. 8.8% (p = 0.01).

**Conclusion:**

A criteria-based audit proved to be a feasible and useful tool in improving diagnosis and management of obstructed labour using available resources. Some of the observed changes in practice were of modest magnitude implying demand for further improvements, while sustaining those already put in place.

## Introduction

Maternal mortality remains a challenge in the post-Millennium Development Goal (MDG) era, especially in developing countries [1–3], where obstructed labour is one of the leading causes of maternal death [4]. Obstructed labour affects 3% to 6% of labouring women globally [5], and in low-resource settings is closely associated with severe maternal morbidity such as postpartum haemorrhage [6,7], uterine rupture [8,9], puerperal sepsis [10], genital fistula [9,11,12] and maternal death [13,14]. Obstructed labour also carries a high risk of intrapartum asphyxia, subsequent neonatal neurological damage, and perinatal death [11,15]. In Tanzania, obstructed labour and its complications has been reported as one of the leading cause of maternal and perinatal mortality [16–18] that is highly associated with substandard obstetric care [10]. Thus, prevention of complications related to obstructed labour should include timely diagnosis, resuscitation, and relief of obstruction, either by caesarean delivery or assisted vaginal delivery, including vacuum extraction.

WHO has defined obstructed labour as the failure of the presenting part of the foetus to progress into the birth canal, despite strong uterine contractions [19]. Notwithstanding the clarity of the definition, obstructed labour can be difficult to predict due to the dynamic nature of the process, including changes in the position of the foetal head that can alter the dimension of the presenting part as it descends in the pelvic canal. Furthermore, risk factors for obstructed labour that may be identified at antenatal care, such as small stature, early marriage, and nulliparity, have not shown sufficient positive predictive value to serve as a screening tool [20,21]. Therefore, optimal clinical diagnosis is essential for prompt management of obstructed labour.

In low-income countries such as Tanzania substandard care during labour contributes to as much as 6% of labour-related maternal deaths [10]. In addition, 30% of perinatal mortality is attributed to intrapartum asphyxia in a large proportion of term deliveries [22]. At the national referral hospital in Tanzania, obstructed labour contributes up to 3% of maternal deaths [16]. In the same setting, recent cross-sectional [23,24] and qualitative studies [25,26] have revealed a significant number of questionable decisions in the case of caesarean sections (CS), delayed pre-operative interventions, too few assisted vaginal deliveries, and inadequate use of partograms as the main reasons for substandard obstetric care. Since half of the CS that were performed were due to obstructed labour (MNH database 2014, unpublished report), then it was important to evaluate and improve standards of diagnosis and management of obstructed labour. Furthermore, in the same settings increased rates of CS were associated with low-risk pregnancies, according to the Robson classification of CS deliveries [27]. Although international and national guidelines for the diagnosis and management of obstructed labour are similar [28,29], the variability of access to healthcare from one facility to another can account for disparities in the implementation of such guidelines [30]. Thus, improvement of substandard emergency obstetric care, including the handling of obstructed labour, requires the regular assessment and implementation of safe, efficient obstetric interventions in accordance with local needs and available resources.

A criteria-based audit is a quality improvement tool that systematically and critically assesses the process, structure and outcome of obstetric care. It also requires that providers adhere to a concise checklist of criteria for quality care [31–33]. The aim of this study was to perform a criteria based audit of the diagnosis and management of obstructed labour at a national referral hospital in a low-resource setting, in order to improve the provision of obstetric care with the available resources.

## Methods

### Study design

We conducted a baseline criteria based audit at Muhimbili National referral Hospital (MNH) in Dar es Salaam, Tanzania, from October 2013 to March 2014. Baseline audit performance was discussed and interventions to improve obstetric care were implemented. Obstetric care was reassessed between July and November 2015. The original audit was based on case files of deliveries that included a partograph and a physician’s diagnosis of obstructed labour in the case of a single foetus in cephalic presentation. Exclusion criteria were premature membrane rupture and/or severe medical conditions such as eclampsia, cardiac disease, and severe anaemia (haemoglobin < 7g/dl, as defined in national maternal and child health guidelines). As part of a data validity check, every 1 to 2 weeks some of the audited cases were randomly selected and their registration numbers compared with cases of obstructed labour in the delivery books and with case files retrieved from medical records.

### Study settings

#### Obstetric care at MNH

The study was carried out at MNH. This hospital receives patients from Dar es Salaam city as well as other parts of the country. Most of the patients come from Pwani region. About 60% of those who come to MNH are self-referred. According to the Tanzania Demographic Health Survey (2010), the average CS rates in Dar es Salaam city and the Pwani region from 2005 to 2010 were 13% and 6%, respectively, above the estimated national CS rate of 4.5%. In 2014 the national referral hospital conducted about 8000 deliveries. The CS rate was 56%, maternal mortality was 313/100,000 live births, stillbirths were 87/1000 live births (of which 45% were fresh stillbirths), and the neonatal distress rate (i.e., Apgar score 1–6 at 5^th^ minute after delivery) was 63/1000 live births. Improvement of the Dar es Salaam referral system without adequately equipping the referring health facilities with adequate tools for comprehensive EmOC has contributed to the disparity of the CS rate and severe morbidity between highest referral point, MNH, and the other hospitals in Dar es Salaam and Pwani region.

The MNH maternity wards are staffed by 25 obstetricians who work with 28 obstetrics and gynaecology residents, 4 registrars, and approximately 25 nurse-midwives. The two obstetric operating rooms are located adjacent to the maternity building, which has 120 beds and comprises antenatal, neonatal, and post-natal wards and a unit for seriously ill patients, including those with eclampsia. MNH has private and public wards with similar management routines, but accommodations in the private wards are more comfortable; patients in the private wards choose their attending specialists and pay for service. The national health policy provides maternity care free of charge.

#### Obstetric database

The main source of obstetric statistics is a database established in 1998 [33]. Information from antenatal care forms and medical records is entered into the maternity book and then computerized. The registry records the date and time of admission, age, parity, referral status, antenatal clinic attendance, reason for admission, time and mode of delivery, indication of CS, and delivery outcome (estimated blood loss, Apgar score, birth weight, sex of baby, and maternal and foetal outcomes). Causes of early neonatal deaths based on clinical diagnosis of the underlying illness can be traced from the neonatal unit records.

#### Delivery room procedures

On admission to the delivery room, all women are seen by a nurse-midwife and a brief history is taken that includes personal data, next of kin, antenatal history, prior obstetrical record, and anticipated risks in the current pregnancy, of which is entered in the partogram. The initial obstetric assessment is routinely done by a resident/registrar but sometimes by the specialist on call. The pelvic assessment of the progress of labour is conducted by the doctor on call. Nurse-midwives perform half-hourly foetal heart rate monitoring by intermittent foetal heart auscultation using the Pinard Fetoscope or the hand-held Fetal Doppler, and also perform vaginal deliveries if there are complications. Available uterotonic available in the delivery room include oxytocin and ergometrine and, occasionally misoprostol. Active management of third stage of labour is mandatorily performed. Women who deliver vaginally without complications are later transferred to the postnatal ward and observed for at least six hours before being discharged from the hospital. Those delivered by CS are given a routine pre-operative assessment and undergo preparations using a checklist that includes the patient’s personal information, indication for CS, signed informed consent, haemoglobin level, blood group and cross-matching, prophylactic antibiotics, a preload of intravenous fluid, catheterization, vital signs, time the CS decision was made, time the patient was taken to the operating theatre, and a nursing intervention report. According to departmental protocol all decisions to proceed with a CS must be made in consultation with, or by, a specialist.

### Development of the audit form

The audit form was developed and pre-tested to capture a patient’s background data and all clearly-defined indicators that emerged in the process of diagnosing and managing obstructed labour. An expert statistician and two senior obstetricians with experience in clinical audits checked the audit form for clarity and relevance in identifying measures of process and outcome in the clinical management of obstructed labour. The revision process included discussions followed by the modification or deletion of inappropriate items. The form was pilot-tested for 30 patients, and the review process continued until the experts, audit evaluators, and data collectors were satisfied that the forms were clear and accurately collected the desired information.

### Audit procedure

**Fig 1:**
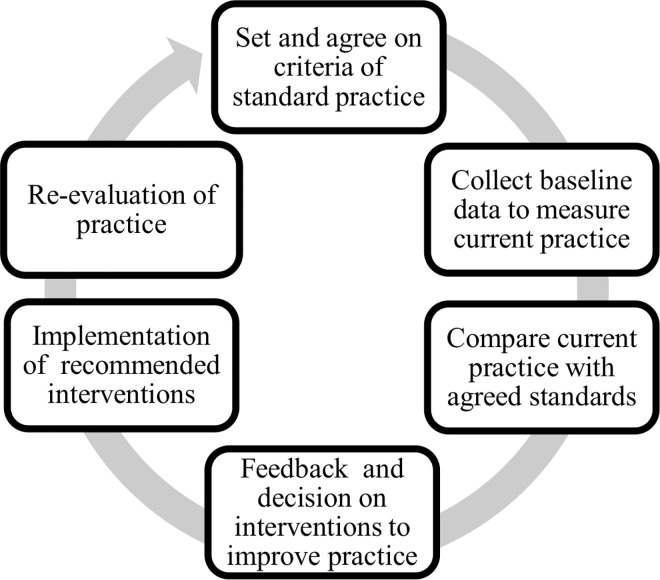
Criteria-based audit cycle.

The criteria based audit procedure included five steps (Fig 1). A clinical audit can be defined as the systematic and critical analysis of the “quality of medical care, including the procedures used for diagnosis and treatment, the use of resources and the resulting outcome, and the quality of life of the patient” [33].

#### Step One: Set and agree on criteria of standard practice

Best practice criteria for the diagnosis and management of obstructed labour were generated after a reviewing a) scientific publications and textbooks, b) WHO manual [28], and c) Tanzania national guidelines [29]. The list of criteria was then reviewed and modified by a panel of four obstetricians, two midwives, and eight obstetric residents. The modified criteria were later discussed and agreed upon in a departmental meeting that included 55 doctors, midwives, anaesthesiologists, pharmacists, laboratory technicians, and maternity ward attendants. Fulfilment of the agreed diagnostic criteria required inclusion of at least one major and one additional minor criterion (Table 1), while management criteria included all those in Table 2. During the discussion, the operationalization of the guidelines by using exact units for the rate of cervical dilatation was thought to be too precise and difficult to assess in the process of collecting data from the partogram. It was, therefore, agreed that the rate of dilatation should be measured in full integers, i.e., 1 to 2 cm/hour, as the partogram was validated by Philpott and Castle [34]. A decision on the final criteria for standard diagnosis and management was reached by consensus, although the most senior member of the group arrived at the final decision if the participants failed to agree.

**Table 1:** Criteria of diagnosis for obstructed labour, agreed upon by consensus.

*Major criteria*
1. Prolonged active labour^a^ of ≥ 8 hours for primiparas and ≥ 6 hours for multiparas,
2. Regular good uterine contractions^b^
*Minor criteria*:
1. Protracted cervical dilatation < 1 cm per hour for primiparas and < 2 cm per hour for multiparas
2. Protracted descent of the fetal head at less than one-fifth per hour in primiparas or less than two-fifths per hour in multiparas
3. Arrested cervical dilatation for > 3 hours for primiparas and > 2 hours for multiparas
4. Arrested descent of the presenting part for > 1 hour for both primiparas and multiparas
5. Prolongation of second stage of labour for > 2 hours for primiparas and > 1 hours in multiparas
6. Presence of severe caput, which implying inability to palpate moulding, or documented caput of ≥ 2+
7. Presence of severe moulding implying documented moulding of 3+

^a^ cervical dilation ≥ 3 cm and regular good uterine contractions

^b^ ≥ 3 contractions in 10 mins, lasting ≥ 20 second per contraction

**Table 2:** List of agreed upon criteria for standard management of obstructed labour at national referral hospital.

*Standard management guidelines*
1. Start intravenous hydration with at least 1 litre of crystalloids (Ringer’s lactate or normal saline)
2. Urinary bladder should be drained by an indwelling urethral catheter
3. Blood typing and cross-matching should be done
4. Broad spectrum antibiotics should be administered (Metronidazole must be included)
5. Informed consent should be obtained from patient
6. Pre-operative checklist should be used to verify management protocol and timelines of intervention from decision to arrival in operating theatre
7. Review by a specialist at least once during process of labour to delivery, either in person, by phone, or during major/service ward rounds
8. Caesarean section should commence within 1 hour after decision to proceed: interval from decision to theatre arrival should be less than 30 mins, and from theatre arrival to delivery should be less than 30 additional minutes.

#### Step Two: Collect baseline data to measure current practice

Current practice was determined by the collection of baseline data on the diagnosis and management of obstructed labour. Trained postnatal ward nurses used a pre-tested audit form to capture a patient’s background as well as indicators that showed the process and management of obstructed labour. If the information was missing in the case files, other sources, including the postnatal ward admissions and report book, theatre analysis record, and interviews with patients, were used to fill in the missing data. Audit evaluators, one consultant, one specialist, and one midwife were available on a daily basis for consultations if there were difficulties understanding the information in the case file and partograph. The audit forms were inspected for missing information before data entry. Data quality control measures included comparison of details in randomly selected audit forms with corresponding information in case files and labour ward analysis book.

#### Step Three: Compare current practice with agreed standards

The baseline of current practice was compared with the agreed upon standards of diagnosis and management of obstructed labour, and the results summarized. The audit evaluators then assessed the fulfilment of the criteria, evaluating the recorded practice against the agreed upon criteria for best practice. In case of disagreement, decisions were based on the consensus of the panel members.

#### Step Four: Feedback and decision on interventions to improve practice

A summary of the analysis of the standards applied in the diagnosis and management of obstructed labour was discussed by 65 stakeholders in 7 groups of 8 to 10 people during a one-day workshop. Participants included obstetricians, obstetric residents and registrars, nurse midwives, maternity ward attendants, anaesthetists, pharmacists, and laboratory technicians. They came from maternity wards at MNH and other Dar es Salaam and Pwani public health facilities. As a result of the workshop, changes in practice were suggested and a summary of each discussion group’s recommendations was presented to the assembled stakeholders for approval (Tables 3 and 4).

**Table 3:** Recommended interventions to improve diagnosis of obstructed labour.

1. Post list of agreed upon criteria for standard diagnosis of obstructed labour in labour ward and operating theatre reception area
2. Midwife in charge and specialist on call should periodically remind doctors to adhere to criteria during grand rounds and routine work
3. Confirm diagnosis of obstructed labour in case log notes according to posted criteria when patient is sent to or received in theatre
4. Promote utilization and interpretation of partogram by regular training on its use during ward rounds
5. Encourage doctors at the referral points to use posted criteria to confirm diagnosis before referring patients because of obstructed labour

**Table 4:** Recommended interventions to improve management of obstructed labour.

I. *Interventions to improve pre-operative assessment and management*
1. Specialist on call should be present within hospital compound at all times.
2. Enforce mandatory documentation of identity of all those who review patients, either in person, over the phone, or on major ward rounds
3. In case of emergency, in the absence of a resident, midwives should communicate directly with a specialist
4. The specialist on call should make regular visits to the labour ward for a minimum of three service rounds a day: morning, afternoon, and evening
5. Ensure availability of a vacuum extractor, and conduct regular retraining of nurses, doctors, residents, and obstetricians in its use
II. *Interventions to improve timely progress from decision to delivery*
1. Incorporate the decision to proceed to delivery as “the Golden 60 Minutes” in the kaizen (Japanese “improvement”) quality improvement system
2. Strengthen teamwork and task sharing between specialists on call, residents, and nurse midwives
3. Enforce mandatory communication from labour room to operating theatre whenever decision to perform CS is made, in order to facilitate prioritization in theatre
4. Institute demand-driven allocation of midwives according to workload, especially during off-hours including night shift and public holidays
5. When assigning shift person-in-charge on labour ward and in obstetric theatre consider leadership abilities of those chosen in order to improve effectiveness during work
6. Patients for CS should be triaged in theatre by obstetrician or resident-on-call, theatre nurse, and anaesthesiologist/anaesthetists for appropriate prioritization.
7. Doctor’s decision to proceed to CS should be accompanied by documentation of level of emergency in order to facilitate prioritization
8. Gynaecological operating theatre should be made available for obstetric patients in case the number of patients waiting for emergency CS overwhelms the capacity of the two obstetric theatres
9. Doctors should refer cases for CS as soon as a decision is made, rather than accumulating a number of several patients and sending them for CS all at once

#### Step five: Implementation of recommended interventions

The recommendations presented to the stakeholders for implementation were posted in the labour room and operating theatres. Similarly, representatives from the referral points agreed upon the implementations and briefed their colleagues. The implementation phase was carried out over a period of four months, from March to June 2015.

#### Step six: Reevaluation of practice

A re-audit was conducted from 1 July to 31 November 2015. The outcome was evaluated by comparing the implementation of the practices agreed upon baseline and to re-audit results by percentage.

### Sampling, measuring instrument, and data collection

Using Epi Info 7, the minimum required sample size was 256 participants for the baseline audit. This assumes that 767 patients were delivered by CS because of obstructed labour at a CS rate of 49% of 10,433 deliveries in 2012 (MNH database 2012, unpublished data). Since the percentage of substandard care in both diagnosis and management was unknown, we assumed the worst case scenario of 50%, with an absolute precision of 5%. We wished to detect a 10% improvement in standard care; hence the post-intervention substandard care was estimated at 40%. Therefore, the minimum sample size required for the re audit was 250 cases.

Data on age, parity, patient referral category, mode of delivery, progress of labour details, and management of obstructed labour, including pre-operative preparations, type and timelines of interventions, and outcome of pregnancy, were collected using a pre-tested form.

The research assistants used a pre-tested form to record the background as well as indicators that showed the process and management of obstructed labour from the case files, other sources when necessary, including the postnatal ward admissions and report book, theatre analysis record, and interviews with patients.

### Participants’ recruitment

Participants included all patients that had a diagnosis of “obstructed labour” and were identified from labour ward birth registry every morning at 08.00 hours for recruitment of patients that delivered the previous night, and every afternoon at 16.00 hours for those that delivered during the day hours of the same day. Participants’ identification was by patient registration number and name. Case files, partograph and antenatal care record were then pulled from the respective wards and were reviewed for patient eligibility before data was collected for the study.

### Statistical analyses

Data was entered and analyzed using SPSS (IBM SPSS, Chicago, IL). We analysed the difference in percentage of those fulfilling ≥ 1 major criterion and ≥ 1 minor criterion for diagnosis and all eight criteria for management at baseline and at re-audit, as well as fulfilment of each major and minor criterion for diagnosis and management using Student’s *t*-test. Difference in median time between decision to perform CS to theatre arrival, between theatre arrival to delivery, and from CS decision to delivery in the baseline and re-audit was analyzed using median test. Differences between baseline audit and re-audit in obstetric history and mode of delivery for substandard diagnosis and management were analyzed using Pearson’s Chi-square test or Fisher’s exact test, as appropriate. The level of significance (α) was at *p* < 0.05.

### Ethical considerations

Ethics approval was obtained from the Muhimbili University of Health Sciences, Research and Publications Committee on 30 July 2013 (letter of reference No. MU.DRP/AEC/Vol. XVI/192). Written informed consent was obtained from each of those participants whom we interviewed about their care in order to qualify unclear information from their case files. The consent form was also approved by the Muhimbili University of Health Sciences, Research and Publications Committee and stipulated that participation to the study was completely voluntary.

## Results

### Standards of diagnosis of obstructed labour in the baseline and re-audit

There was a significant increase in the percentage of women whose intake fulfilled agreed criteria for a standard *diagnosis* of obstructed labour in the re-audit, compared to the baseline audit (74% vs. 81%; *p* = 0.049) (Table 5). The change was partly due to significant improvement in the detection and recording of major criteria, including prolonged labour (22% vs. 38%; *p* < 0.001) and regular uterine contractions (68% vs. 76%; *p* = 0.036); and minor criteria including protraction of dilation and descent, arrested dilatation, and severe moulding (all *p* < 0.04). There were also reduced proportions of cases that were diagnosed as obstructed labour out of all deliveries from 11% (260 cases/2405 deliveries) to 7.2% (250 cases/3462 deliveries)(p<0.001)

**Table 5:** Percentage of cases fulfilling criteria for diagnosis at baseline and re-audit including *p*-value for *t*-test of difference.

*Standards*	*Criteria for diagnosis of obstructed labour*						
	Baseline audit (n = 260)			Re-audit (n = 250)			*p*-value
	n	Missing	%	n	Missing	%	
Fulfilled: ≥ 1 major and ≥ 1 minor criteria	191/260	0	73.5%	202/250	0	80.8%	0.049
*Fulfilled major criteria*							
Prolonged labour	57/260	88	21.9%	96/250	65	38.4%	< 0.001
Regular uterine contractions	177/260	7	68.1%	191/250	2	76.4%	0.036
*Fulfilled minor criteria*							
Protraction of dilation	36/260	60	13.9%	68/250	52	27.2%	< 0.001
Protraction of descent	40/260	60	15.4%	63/250	53	25.2%	0.006
Arrested dilation	34/260	60	13.1%	67/250	55	26.8%	< 0.001
Arrested descent	74/260	60	28.5%	72/250	54	28.8%	0.93
Prolonged second stage	80/260	51	30.8%	71/250	53	28.4%	0.56
Severe caput	112/260	21	43.1%	115/250	6	46.0%	0.51
Severe moulding	53/260	55	21.2%	92/250	23	36.8%	< 0.001

### Standards of management of obstructed labour in the baseline and re-audit

There was also a significant increase in the percentage of cases that satisfied criteria for standard management of obstructed labour, when comparing baseline and re-audit (4.2% vs. 9.2%; *p* = 0.025) (Table 6). The improved management statistics were in part attributable to a significant increase in meeting the review by obstetrician criteria (34% vs. 43%; *p* = 0.046). Regardless of the subsequent mode of delivery, there was increased adherence to the prescribed time interval from decision to proceed with CS to delivery (15% vs. 20%; *p* = 0.14). However, when the analysis was limited to women delivered by CS, there was significantly increased adherence to time interval from decision of CS to delivery (10% vs. 17%: *p* = 0.023). This was due to an increase in both adherence to the time interval from decision to proceed with CS to the patient’s arrival at the operating theatre, and from arrival to theatre to being operated upon. On average, the improved timeline of the intervention resulted from reducing the total decision-to-delivery time by 30 minutes (Table 7), a result of shortening the decision to theatre arrival interval and the theatre arrival to delivery interval (all *p* < 0.001)

**Table 6:** Percentage of cases fulfilling improved criteria for management of obstructed labour at baseline and re-audit including *p*—value for Student’s *t*-test of difference.

*Standards*	*Criteria for management of obstructed labour*						
	Baseline audit (n = 260)			Re-audit (n = 250)			*p*-value
	n	Missing	%	n	Missing	%	
Fulfilled all of criteria	11/260	0	4.2%	23/250	0	9.2%	0.025
*Fulfilled criteria*							
Intravenous fluids resuscitation	251/260	4	96.5%	245/250	4	98.0%	0.31
Pre-operative prophylactic antibiotics	253/260	1	97.3%	233/250	10	93.2%	0.029
Urethral catheterization	254/260	4	97.7%	242/250	4	96.8%	0.54
Blood grouping and X matching	257/260	0	98.8%	248/249	1	99.6%	0.34
Reviewed by a senior	89/260	0	34.2%	108/250	0	43.2%	0.045
Informed consent	255/260	0	98.1%	246/250	0	98.4%	0.78
Lack of preoperative check list	251/260	0	96.5%	239/250	0	95.6%	0.59
Decision delivery interval	40/260	0	15.4%	51/250	0	20.4%	0.14
*Timeline for patients delivered by CS*							
Decision-to-delivery (≤ 60 min)	24/240	0	10.0%	41/240	0	17.1%	0.023
Decision-to-theatre(≤ 30 min)	56/240	0	23.3%	84/240	0	35.0%	0.005
Theatre-to-delivery(≤ 30 min)	39/240	0	16.3%	72/240	0	30.0%	< 0.001

**Table 7:** Median (range) time (minutes) between baseline and re-audit in cases delivered by CS.

*Timeline of intervention*	*Median (range) time interval*		
	Baseline audit	Re-audit	*p*-value
From decision to delivery	120 (20–852)	90 (40–379)	< 0.001
From decision to theatre	55 (7–255)	42 (10–137)	< 0.001
From theatre to delivery	60 (10–720)	45 (13–309)	< 0.001

### Substandard care based on background factors in the baseline and re-audit

There was a reduction in substandard care in the management of parturients ≥ 35years old (100% vs. 80%; *p* = 0.003), and parity 2 to 4 (95% vs. 87%; *p* = 0.036) in the re-audit, compared to the baseline audit (Table 8). Referred women had less substandard diagnoses (32% vs. 22%; *p* = 0.029) and management (98% vs. 93%; *p* = 0.010) in the re-audit, compared to the baseline audit. In addition, care of referred patients in the public category had improved both diagnosis (33% vs. 21%; *p* = 0.016) and management (99% vs. 93%; *p* = 0.005).

**Table 8:** Percentage of cases with substandard diagnosis and management at baseline (n = 260) and re-audit (n = 250) by obstetric history and patient category including *p*-value for *t*-test of difference.

*Characteristic*	*Substandard diagnosis*			*Substandard management*		
	Baseline audit	Re-audit	*p*-value	Baseline audit	Re-audit	*p*-value
	(%)	(%)		(%)	(%)	
*Maternal age (yrs)*						
< 20	33.3	12.0	0.068	100	96.0	0.48
20–34	25.0	21.7	0.460	94.3	92.4	0.47
≥ 35	29.2	12.1	0.057	100	80.5	0.003
*Parity*						
1	26.4	18,4	0.100	95.6	93.9	0.41
2–4	27.5	19.1	0.160	95.4	87.2	0.036
≥ 5	20.0	33.3	0.640	100	77.8	0.13
*Gestational age (wks)*						
< 37	34.4	28.5	1.000	100	85.7	0.069
37–42	25.0	18.6.1	0.100	94.8	90.9	0.11
≥ 43	30.0	20.0	1.000	100	100	1.0
*Source of admission*						
Referred	31.8	21.7	0.029	98.4	93.3	0.01
Non-referred	9.7	12.6	0.590	87.1	84.5	0.67
*Payment category*						
Public	31.4	21.4	0.029	97.5	93.1	0.044
Private	11.1	14.3	0.580	90.5	85.7	0.39
*Payment and referral category*						
Public referrals	32.7	21.4	0.016	98.9	93.1	0.005
Public non-referrals	14.3	0/0	n/a^ɑ^	78.6	0/0	n/a^ɑ^
Private referrals	20.0	33.3	0.600	93.3	100	1.0
Private no-referrals	8.3	12.7	0.460	89.6	84.5	0.60

^ɑ^n/a = not applicable

### Maternal and perinatal outcome in the baseline and re-audit

The rate of CS increased (90% vs 94%; p = 0.18), while that of vacuum extraction (3.5% vs. 3.2%; p = 0.86) and vaginal deliveries (6.2% vs. 3.2; p = 0.11) decreased without significant difference from the baseline compared to re-audit. Similarly, the rate of severe maternal morbidity including postpartum haemorrhage and uterine rupture was comparable between the baseline audit and re-audit (9.0% vs. 8.8%; p = 0.98.) There was only one case of intraoperatively diagnosed uterine rupture during the baseline audit but none during re-audit. None of the study participants were admitted in the intensive care unit during baseline audit and re-audit. The percentage of perinatal severe morbidities and deaths including neonatal distress (i.e., Apgar score 1–6 at 5^th^ minute after delivery) and fresh stillbirths was significantly reduced from the baseline to re-audit (16% vs. 8.8%; p = 0.01).

## Discussion

The criteria based audit served to improve the diagnosis and management of obstructed labour using available resources. The improved diagnosis was achieved by upgrading the skills of practitioners in identifying and recording signs of prolonged labour, intensity of uterine contractions, progress in cervical dilatation, descent of presenting part, and severe moulding. Increased patient review by an obstetrician, and adherence to an agreed upon timeline of intervention from decision to delivery by CS; independently improved care, especially among referred and non-paying patients. It proved the feasibility of using criteria based audit to improve obstetric care in a low resource setting.

Several studies have shown that the prevailing substandard diagnosis in a majority of CS cases leads to a continued overuse of the procedure [23,27]. Improvement in standards of diagnosis have increased the opportunity for timely management and reduced complications of obstructed labour. A two-fold increase in standards of management was noted, but only in 10% of all cases. This confirmed continuing high degree of substandard emergency obstetric care (EmOC) of up to 90% that also prevails in other low resource settings [9,12,35,36]. The persistence of substandard care in both diagnosis and management after our interventions indicates that there is opportunity for improvement if stakeholders sustain their commitment to do better [31]. On the other hand, a lack of improvement in management may be the result of lax criteria and inadequate interventions. Hence, re-evaluation of the criteria adopted as best practices, and an ongoing critical analysis of interventions already put in place are needed for development of continually improving strategies.

The mechanism of labour and delivery process makes prediction of obstructed labour difficult, and also present challenges to accurate detection of obstructed labour. The principally agreed-upon criterion for diagnosis is a prolonged active labour, implying a failure of labour to progress due to cephalopelvic disproportion [15] that requires surgical or assisted vaginal delivery [5]. Maaloe et al. [12] and Kidanto et al. [37] reported on failure to interpret signs of prolonged labour as obstructed labour in both rural and urban health facilities. Unlike the Malawi audit [9], the agreed-upon diagnosis in our study included additional minor criteria that improved the standards of diagnosis. These criteria included evidence-based [38] details of the partographic assessment of progress of labour such as uterine contractions, cervical dilatation, descent of presenting part, and degree of moulding and caput. Therefore, the agreed-upon criteria of best practice for standard diagnosis were not only for operationalization of research, but could also be utilized as clinical management guidelines–something that was absent before introducing the criteria based audit. Improved standards of diagnosis give care providers increased ability to interpret and record their observations in the partogram. The latter was one of the most important changes instituted as a result of in-house training and has improved teamwork among staff in the delivery room. Further, the demonstrated improved diagnosis in our study aligns with the significant reduction of cases diagnosed as obstructed labour from 11%, in the baseline audit, to 7.5% in the re-audit; and also, decreased rate of vaginal deliveries of case with physician diagnosis of obstructed labour from baseline (6.2%) to re-audit (3.2%).Thus a testimony for improved clinical acumen of truly diagnosing obstructed labour.

As others have confirmed, a mandatory patient review by a senior obstetrician and a more efficient timeline for intervention, either singly or in combination resulted in improved care [37,39,40]. However, awareness of care providers that an evaluation was being conducted might also have positively or negatively influenced the changes in practice that were recorded [41]. Involving senior doctors to participate in the patient management strengthens a) leadership with regard to management, b) cooperation with the audit, c) communication, as well as d) facilitating joint decision-making in patient care by seeking a second opinion. Practice based on collective responsibility relieves junior doctors from the fear and blame associated with poor outcomes [42], preventing the practice of defensive medicine [41], and encouraging the use of procedures for decision making and management of EmOC

We incorporated care providers in establishing local standards; so that their involvement early on would give them a stake in the successful implementation and sustainability of the improved care standards. Natural resistance to changes in practice [43] and the danger that dysfunctional teamwork may persist between senior and junior doctors and nurses [42] might have hampered mandatory senior reviews in more than 50% of the cases we examined. Furthermore, unfulfilled staff recommendations, including the provision of comfortable lounge for on-call doctors in proximity to the delivery room and operating theatre might have also minimized the time senior doctors spent with patients resulting in fewer occasions for senior review, and decreasing team work between juniors and seniors.

WHO recommends timely, accessible, and adequate health care as a human right [44]. In the local context in Tanzania timely care is one of the major determinants of severe maternal morbidity [16,45]. Audit intervention according to the agreed standards shortened decision-to-delivery intervals by 30 minutes. Contrary to our agreed-upon optimal one hour cut-off limit from decision to delivery, other studies adopted a two to three hour cut-off limit [9,23,46]. Kongnyuy et al. [9] and Wagaarachchi et al. [46] suggested quality improvement that included primary health care facilities where patients with complications are quickly diagnosed and sent to the top of the health facility ladder, thus lowering the risk of complications associated with delays in receiving needed speciality care. Similarly, Kidanto et al. [37] recommended a decision-to-delivery interval of two hours for eclampsia patients who required resuscitation to prevent surgical complications, including control of seizures and blood pressure levels before CS. Since the majority of women delivered at MNH are either public or referred patients [47], some presented with complications of obstructed labour on admission, including foetal distress, impending or ruptured uterus, and chrorioamnionitis (MNH Obstetric database 2014, unpublished report). Our timeline of intervention criterion had to be relatively stringent in order to promptly care for public and referral cases that would otherwise face the risk of delayed access to care and thereby increased danger of severe morbidity. [45]

The use of gynaecological theatre for obstetric cases has shortened the decision-to-delivery interval by reducing theatre-to-delivery time. However, the relatively long distance from the maternity ward to the gynaecology theatre at MNH (300 meters), has negated some of the time gain from decision-to-theatre, as it takes aides a considerable amount of time to wheel patients to the gynaecological theatre after a decision for CS is made. Moreover, the workload on the maternity ward is dependent on seasonal variation in the rate of admissions, especially in the cases of referred patients. The majority of baseline audit data was collected in the low season (August to January), while re-audit data was mostly gathered in high season (February to July) (MNH obstetric database, unpublished report). Thus, if there is an increased patient management workload during the re-audit, but without a proportional increase in recourses, there may be a limited improvement in care.

Unlike other studies [9,10], our audit interventions succeeded in improving both standards of diagnosis and management, two aspects of obstetric practice that complement each other as predictors of delivery outcomes. In this study, the improved standards of diagnosis and management were associated with significant reduction of rates of neonatal distress and fresh stillbirths following audit interventions. Despite comparability of the maternal outcomes between the baseline audit and re-audit, the reduced vaginal deliveries from the baseline audit compared to the re-audit aligns with increased clinical acumen of diagnosis that might have reduced neglect of cases of obstructed labour that could be among those delivering vaginally but with severe maternal and perinatal morbidities. Alternatively, since the increased clinical acumen of diagnosis implied increased standards in detection of obstructed labour, then the reduced rates vaginal deliveries could be associated with decreased misdiagnosed cases of obstructed labour that usually delivered vaginally during pre-operative preparation or in the operating theatre. Similar to previous studies (23,27) the increased rate of CS and reduced rates of vacuum extraction from the baseline audit compared to re-audit could be an adverse effect of audit resulting from care providers’ anxiety of being evaluated; and hence defensive practise that favours the care providers assessment than patients safety as shown in previous study (41, 42).

One strength of our study was the use of a piloted audit form that improved the relevance of items on which it focuses. The systematic revision of the audit form during piloting increased the clarity of its questions and the reliability of the results. The training of data collectors, the incorporation of regular checking and thereby taking into account of missing cases, and the evaluation and filling-in missing data improved the validity and reliability of the results [48]. Although the consensus of the care providers was needed to approve the list of criteria and interventions, the majority of practices agreed to as standard care had universal validity by conforming to WHO, as well as the International Federation of Gynaecologists and Obstetricians (FIGO), and national standards. Our study’s limitations included the risk of an unrealistic evaluation of care because it was solely based on hospital files. It would have been desirable to assess the actual clinical situations, that is pre- and post-interventions in the delivery room and operating theatre, and changes made in the number of staff on each shift during a 24 hours cycle. The condition of patients before and after interventions might also have changed thereby reducing the reliability of the results.

Improving obstetric care through adherence to an agreed-upon routine reduced negative new-born outcomes. Further analysis of trends of maternal and perinatal outcome is recommended, so that the impact of audit intervention can be revealed overtime rather than at one point in time. Additionally, assessment of the impact of audit interventions on delivery outcomes based on obstetric characteristics of the studied groups (such as in Robson classification) can provide a deeper understanding as to whether the increased of rate of CS was justified or not. Positive obstetric outcomes will validate the interventions that were put in place, and at the same time raise the confidence of care providers and strengthen their commitment to do better [49]. Finally, in-house monitoring and updating of clinical guidelines should be a priority in order to provide sustainable, evidence-based care to mothers and their the new-born

## Conclusion

The CBA proved to be a feasible and useful a tool in improving diagnosis and management of obstructed labour using available resources. Some of the observed changes in practice were of modest magnitude implying demand for further improvements, while sustaining those already put in place. Further improvement of quality of care require an ongoing commitment to do better, in addition to regular audits and feedback to evaluate the process of care and determine what works and what does not.
